# Plasma osteoprotegerin is related to carotid and peripheral arterial disease, but not to myocardial ischemia in type 2 diabetes mellitus

**DOI:** 10.1186/1475-2840-10-76

**Published:** 2011-08-12

**Authors:** Mikael K Poulsen, Mads Nybo, Jordi Dahl, Susanne Hosbond, Tina S Poulsen, Allan Johansen, Poul F Høilund-Carlsen, Henning Beck-Nielsen, Lars M Rasmussen, Jan E Henriksen

**Affiliations:** 1 Dept. of Endocrinology, Diabetes Research Center, Odense University Hospital, Odense, Denmark; 2 Dept. of Biochemistry, Pharmacology and Genetics, Odense University Hospital, Odense, Denmark; 3 Dept. of Cardiology, Odense University Hospital, Odense, Denmark; 4 Dept. of Nuclear Medicine, Odense University Hospital, Odense, Denmark

## Abstract

**Background:**

Cardiovascular disease (CVD) is frequent in type 2 diabetes mellitus patients due to accelerated atherosclerosis. Plasma osteoprotegerin (OPG) has evolved as a biomarker for CVD. We examined the relationship between plasma OPG levels and different CVD manifestations in type 2 diabetes.

**Methods:**

Type 2 diabetes patients without known CVD referred consecutively to a diabetes clinic for the first time (n = 305, aged: 58.6 ± 11.3 years, diabetes duration: 4.5 ± 5.3 years) were screened for carotid arterial disease, peripheral arterial disease, and myocardial ischemia by means of carotid artery ultrasonography, peripheral ankle and toe systolic blood pressure measurements, and myocardial perfusion scintigraphy (MPS). In addition, plasma OPG concentrations and other CVD-related markers were measured.

**Results:**

The prevalence of carotid arterial disease, peripheral arterial disease, and myocardial ischemia was 42%, 15%, and 30%, respectively. Plasma OPG was significantly increased in patients with carotid and peripheral arterial disease compared to patients without (p < 0.001, respectively), however, this was not the case for patients with myocardial ischemia versus those without (p = 0.71). When adjusted for age, HbA1c and U-albumin creatinine ratio in a multivariate logistic regression analysis, plasma OPG remained strongly associated with carotid arterial disease (adjusted OR: 2.12; 95% CI: 1.22-3.67; p = 0.008), but not with peripheral arterial disease or myocardial ischemia.

**Conclusions:**

Increased plasma OPG concentration is associated with carotid and peripheral arterial disease in patients with type 2 diabetes, whereas no relation is observed with respect to myocardial ischemia on MPS. The reason for this discrepancy is unknown.

**Trial registration number:**

at http://www.clinicaltrial.gov: NCT00298844

## Background

Cardiovascular disease (CVD) is the leading cause of death in type 2 diabetes mellitus patients [1,2], as many suffer from coronary heart disease, cerebrovascular disease, and/or peripheral arterial disease (PAD) [3-5]. Even, asymptomatic patients with type 2 diabetes display subtle CVD [6-8], and therefore it has been debated if screening for unknown CVD should be performed in these patients [8].

Osteoprotegerin (OPG) is a soluble member of the TNF-receptor superfamily and act as decoy receptor for both the receptor activator of nuclear factor-B ligand (RANK-L) and the TNF-related apoptosis inducing ligand (TRAIL), two cytokines of the TNF-family [9]. OPG is found in bone, but also other mesenchymal tissue, and the tissue concentration of OPG in aorta and hip-bone are almost equal and approximately 500 times higher than in plasma [10]. The function of OPG in the arterial wall is not known, but it has been suggested that the molecule acts as a vascular calcification inhibitor due to the fact that OPG knock-out mice develop arterial calcifications [11]. Interestingly, we have previously observed significantly higher concentrations of OPG in arteries from patients with diabetes compared to non-diabetics [10]. During recent years, plasma OPG has been attempted used as a biomarker for CVD, at least in some populations [12]. The pathophysiological connection between plasma OPG concentrations and CVD is not known, but relations to both arterial disease as well as to diseased myocardium has been suggested [13,14].

To investigate the relationship between plasma OPG levels and CVD manifestations in type 2 diabetes, all patients were screened for carotid arterial disease, peripheral arterial disease, and myocardial ischemia. Subsequently, we assessed the relationship between these CVD manifestations and plasma OPG.

## Methods

### Patient cohort

We consecutively evaluated all 753 type 2 diabetes patients referred to the Diabetes Clinic at Odense University Hospital, Denmark, from January 2006 to December 2007 of which 305 patients met the inclusion and exclusion criteria as previously reported [15]. The diagnosis of type 2 diabetes was made by each patient's general practitioner using the WHO criteria [16].

The study was carried out according to Good Clinical Practice, followed the Helsinki II Declaration, was approved by the Local Ethics Committee, and registered at http://www.clinicaltrials.gov. All participants gave written, informed consent.

All patients went through a single-day structured examination program including medical history recording and a cardiovascular risk factor evaluation. Physical and laboratory examinations were done and fasting blood samples were obtained for laboratory testing. Finally, patients were screened for CVD in the three vascular territories, i.e., for carotid vessel wall changes using B-mode ultrasound, for PAD using ankle and toe systolic blood pressure measurements, and for myocardial ischemia using myocardial perfusion scintigraphy (MPS).

### Laboratory analyses

Plasma osteoprotegerin concentrations were measured in EDTA-plasma using an immunoassay based on a commercially available sandwich ELISA (R&D Systems, Minneapolis, MN, USA) with specific antibodies against human OPG and europium-labeled streptavidin for detection. Bound europium was measured by time-resolved fluorometric detection using an Autodelfia Instrument from Perkin Elmer/Wallac (Turku, Finland). The analytical range was 62.5-20000 ng/l with an intra-assay imprecision of 4% and an inter-assay imprecision of 7%.

HbA1c was measured by cation-exchange chromatography using Tosoh G7 (Medinor, Broendby, Denmark) with dedicated reagents. Glucose, total cholesterol, LDL cholesterol, HDL cholesterol, and high-sensitive CRP were all analyzed on a Modular Analytics P (Roche Diagnostics, Switzerland) with methods applied as recommended by the supplier.

In a morning spot urine sample U-albumin was measured using immunoturbidimetry (Roche, Basel, Switzerland). The discrimination limit for U-albumin was < 10 mg/l. In the case of U-albumin < 10 mg/l the U-albumin creatinine ratio was set to 0.

### B-mode ultrasound scans of the carotid artery

The B-mode ultrasound scans of the carotid arteries were performed using a Vivid-5 GE medical ultrasound machine (10-MHz linear transducer) and continuous ECG recording during the procedure. The carotid intima-media thickness (CIMT) was measured semi-automatically as the mean of minimum 200 measurements of CIMT per location site from digitally stored images. CIMT was measured bilaterally at the posterior wall (at the bulbus region and at the proximal end of the common carotid artery), using two independent images at end-diastole. Each image was also investigated for plaques using the ARIC-study plaque definition [17]. Carotid arterial disease was defined as mean CIMT > 1.00 mm [18] and/or the presence of a plaque at any carotid location. The limits of agreement between two observers (AJ, MKP) in 20 randomly selected patients on mean CIMT was: average = 0.039 ± 0.061 mm (95% limits of agreement: -0.158-0.081). The absolute interobserver agreement on plaques was 98% (kappa: 0.74).

### Ankle and toe systolic blood pressure measurements

Ankle and toe systolic blood pressure measurements were performed using the strain gauge technique [19]. All subjects rested for 20 min. in the supine position before cuffs were placed around the ankles (cuff width: 12.0 cm) and proximal phalanges of the big toe (cuff width: 2.5 cm). Strain gauges were placed around the distal phalanges of the big toe. Duplicate measurements of ankle and toe systolic blood pressures were obtained. Arm systolic and diastolic blood pressures were measured. The ankle-brachial index (ABI) and the toe systolic blood pressure index (TSPI) were calculated as the systolic blood pressure at each level, respectively, divided by the arm systolic blood pressure. PAD was defined as ABI < 0.90 and/or TSPI < 0.64.

### Myocardial perfusion scintigraphy

Myocardial perfusion scintigraphy (MPS) examinations were performed using stress ECG-gated ^99 m^technetium MPS according to standards of the American Society of Nuclear Cardiology using ECG-gated single photon emission computed tomography [20]. The MPS protocol used in the present population has been published previously [15]. In brief, whenever a potential stress-induced perfusion defect was observed, an additional rest study was carried out to evaluate the degree of reversibility. A semi-quantitative visual interpretation was made by means of short axis, horizontal-, and vertical long axis myocardial tomograms and a 20-segment model. All images were analyzed by two experts (AJ, PFHC) blinded to all other data and each other, and a summed stress score (SSS) was calculated. Myocardial ischemia was defined as a regional perfusion abnormality with a total SSS ≥ 4 and at least one segment having a SSS ≥ 2 [15].

### Statistics

Continuous variables are presented as mean and standard deviations and categorical variables as numbers, and percentages with 95% confidence intervals (CI). Student's t-test was used to test for differences between independent continuous variables. Due to a non-Gaussian distribution of U-albumin creatinine ratio, hs-CRP and plasma OPG, these parameters are presented as median and interquartile range, and the Mann-Whitney test was used to compare groups. The χ^2^-test was used to test for differences between categorical variables. Univariate and multivariate logistic regression analysis were performed, using carotid arterial disease, peripheral arterial disease, and myocardial ischemia as separate outcome variables. The co-variables were adjusted for age, HbA1c and U-albumin creatinine ratio. Results are reported as odds ratio (OR) with 95% CI and p-values. A p-value < 0.05 was considered statistically significant. STATA version 9.2 was used for calculations.

## Results

Characteristics of the patients are presented in Table 1. The prevalence of CVD in the present study has been published previously [15].

**Table 1:** Characteristics of the type 2 diabetes patients

	Total	CVD by examination	No CVD by examination
	(n = 305)	(n = 183)	(n = 122)
Females/males	139/166	77/106	62/60
Age (years)	58.6 ± 11.3	60.7 ± 11.1 *††*	55.3 ± 11.0
Diabetes duration (years)	4.5 ± 5.3	4.7 ± 5.5	4.1 ± 4.9
BMI (kg/m^2^)	32.2 ± 5.8	32.3 ± 6.3	32.2 ± 5.0
Waist circumference (cm)	107.9 ± 14.3	108.2 ± 15.0	107.5 ± 13.1
Hip circumference (cm)	110.2 ± 11.5	110.3 ± 12.3	110.2 ± 10.2
Blood pressure, systolic (mmHg)	138.7 ± 18.1	140.1 ± 17.0	136.6 ± 19.6
Blood pressure, diastolic (mmHg)	79.5 ± 10.9	79.3 ± 10.4	79.8 ± 11.7
***Antidiabetic medical treatment***			
No antidiabetic treatment	65 (21)	40 (22)	25 (20)
Metformine	189 (62)	107 (58)	82 (67)
Rosiglitazone	19 (6)	14 (8)	5 (4)
DPP-IV-inhibitor	5 (2)	4 (2)	1 (1)
Sulphonylurea	76 (25)	46 (25)	30 (25)
Insulin	73 (24)	46 (25)	27 (22)
***Blood samples/urine samples***			
Fasting p-glucose (mmol/l)	8.6 ± 2.5	8.8 ± 2.5	8.4 ± 2.4
HbA1c (%)	7.3 ± 1.3	7.5 ± 1.3 *†*	7.1 ± 1.2
Fasting C-peptide (pmol/l)	1146 ± 644	1169 ± 688	1112 ± 573
Fasting insulin (pmol/l)	106 ± 104	112 ± 119	96 ± 77
Total cholesterol conc. (mmol/l)	4.4 ± 1.0	4.4 ± 1.1	4.3 ± 0.9
LDL-cholesterol conc. (mmol/l)	2.2 ± 0.8	2.2 ± 0.9	2.1 ± 0.7
HDL-cholesterol conc. (mmol/l)	1.28 ± 0.34	1.29 ± 0.34	1.26 ± 0.35
Triglycerides (mmol/l)	2.07 ± 1.29	2.06 ± 1.31	2.09 ± 1.27
Creatinine (μmol/l)	90 ± 24	91 ± 24	88 ± 25
U-albumin creatinine ratio (mg/mmol)*	0.8 (0.0-3.5)	0.8 (0.0-3.1)	0.8 (0.0-3.8)
Hs-CRP (mg/l)*	2.9 (1.4-6.9)	3.0 (1.3-6.8)	2.8 (1.6-7.2)
Osteoprotegerin (μg/l)*	1.11 (0.88-1.46)	1.22 (0.94-1.54) *††*	1.05 (0.78-1.24)
***Risk factors for CVD***			
Family history of CVD*^#^*	58 (19)	31 (17)	26 (21)
Hypertension^##^	197 (64)	124 (68)	72 (59)
Hypercholesterolemia^##^	193 (63)	113 (62)	80 (66)
Current smoker	82 (27)	49 (27)	32 (26)
***Angina pectoris***			
None	236 (77)	139 (76)	97 (80)
Non-cardiac chest pain	40 (13)	24 (13)	16 (13)
Atypical angina pectoris	17 (6)	11 (6)	6 (5)
Typical angina pectoris	12 (4)	9 (5)	3 (2)

Data are means ± SD, (*median (interquartile range)) or n (%). CVD by examination = cardiovascular disease obtained from B-mode ultrasound scans of the carotid arteries, ankle and toe systolic blood pressure measurements and/or myocardial perfusion scintigraphy.

Patients with CVD vs. without CVD by examination: † = p < 0.05. †† = p < 0.01.

DDP-IV-inhibitor = Dipeptidyl peptidase IV inhibitor treatment. See text for definitions.

^**#**^A positive family history of cardiovascular disease defined as cardiovascular disease either in male first-degree relative < 55 years or in female first-degree relative < 65 years.

^**##**^A medical history of hypertension or hypercholesterolemia, respectively.

* tested with the Mann-Whitney nonparametric test.

**Figure 1:**
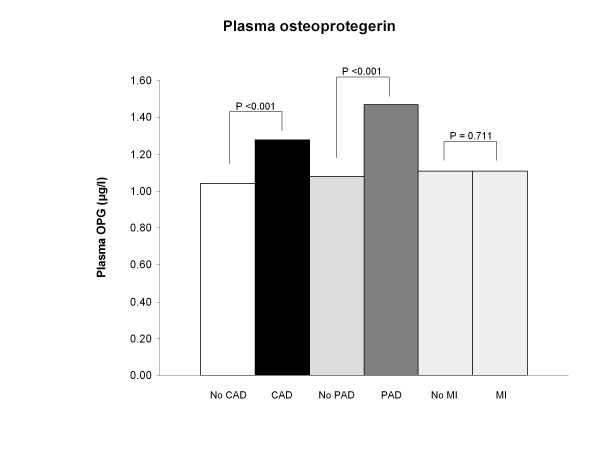
**Plasma osteoprotegerin in patients with and without carotid or peripheral arterial disease or myocardial ischemia**. OPG = osteoprotegerin. CAD = carotid arterial disease. PAD = peripheral arterial disease. MI = myocardial ischemia on MPS.

The plasma OPG concentrations were significantly increased in patients with carotid arterial disease and PAD compared to patients without (1.28 (0.99-1.61) versus 1.04 (0.80-1.33) μg/l, p < 0.001 and 1.47 (1.02-1.89) versus 1.08 (0.85-1.40) μg/l, p < 0.001, respectively) (Table 2 and Figure 1). However, this was not the case for patients with myocardial ischemia versus those without myocardial ischemia (1.11 (0.87-1.55) versus 1.11 (0.89-1.43) μg/l, p = 0.711) (Table 2 and Figure 1). Furthermore, the upper quartile of plasma OPG and myocardial ischemia were tested in a univariate logistic regression analysis and did not show any relationship (OR: 1.46; 95% CI: 0.85-2.53; p = 0.172). CIMT was correlated with plasma OPG concentrations in a simple linear correlation analysis (r = 0.249; p < 0.001). Plasma OPG concentrations were associated with carotid arterial disease in both univariate and multivariate logistic regression analysis when adjusted for age, HbA1c and U-albumin creatinine ratio (adjusted OR: 2.12; 95% CI: 1.22-3.67; p = 0.008), but not peripheral arterial disease or myocardial ischemia (adjusted OR: 1.77; 95% CI: 0.97-3.22; p = 0.063 or adjusted OR: 1.26; 95% CI: 0.77-2.06; p = 0.364, respectively) (Table 3). Systolic blood pressure was significantly increased in patients with carotid arterial disease compared to those without (141 ± 17 versus 137 ± 19 mmHg, p = 0.028). However, systolic blood pressure was not significantly different in patients with or without PAD or myocardial ischemia, respectively. Further diastolic blood pressure was significantly increased in patients with PAD compared to those without (80 ± 11 versus 76 ± 11 mmHg, p = 0.033). Diastolic blood pressure was on the other hand not different in patients with or without carotid arterial disease or myocardial ischemia, respectively (Table 2). In the multivariate logistic regression analysis only systolic blood pressures was associated with carotid arterial disease, but not with PAD or myocardial ischemia, respectively (Table 3). HbA1c and U-albumin creatinine ratio were not significantly differently in patients with or without carotid arterial disease, PAD or myocardial ischemia, respectively (Table 2). Finally, total cholesterol, LDL cholesterol, HDL cholesterol, triglyceride and high-sensitive C-reactive protein (hs-CRP) concentrations were not significantly different or associated with carotid arterial disease, PAD, or myocardial ischemia, respectively (Table 2 and 3).

**Table 2:** Comparison of risk factors of cardiovascular disease and different cardiovascular disease manifestations

	Carotid arterial disease	No carotid arterial disease	
Co-variables	(n = 129)	(n = 176)	p
Blood pressure, systolic (mmHg)	137 ± 19	141 ± 17	0.028
Blood pressure, diastolic (mmHg)	80 ± 11	79 ± 11	0.649
HbA1c (%)	7.3 ± 1.2	7.3 ± 1.3	0.851
U-albumin creatinine ratio (mg/mmol)*	0.6 (0.0-2.8)	1.0 (0.0-4.3)	0.467
Total cholesterol (mmol/l)	4.4 ± 1.2	4.4 ± 0.9	0.861
LDL cholesterol (mmol/l)	2.2 ± 0.9	2.2 ± 0.8	0.930
HDL cholesterol (mmol/l)	1.31 ± 0.36	1.26 ± 0.33	0.193
Triglycerides (mmol/l)	1.97 ± 0.11	2.15 ± 0.10	0.236
Hs-CRP (mg/l)*	2.6 (1.3-6.3)	3.0 (1.7-7.5)	0.306
Osteoprotegerin (μg/l)*	1.28 (0.99-1.61)	1.04 (0.80-1.33)	< 0.001

	**Peripheral arterial disease**	**No peripheral arterial disease**	
**Co-variables**	**(n = 45)**	**(n = 260)**	**P**

Blood pressure, systolic (mmHg)	138 ± 18	143 ± 19	0.064
Blood pressure, diastolic (mmHg)	80 ± 11	76 ± 11	0.033
HbA1c (%)	7.3 ± 1.3	7.6 ± 1.2	0.084
U-albumin creatinine ratio (mg/mmol)*	0.6 (0.0-19.9)	0.8 (0.0-3.0)	0.098
Total cholesterol (mmol/l)	4.4 ± 1.2	4.4 ± 1.0	0.930
LDL cholesterol (mmol/l)	2.1 ± 0.9	2.2 ± 0.8	0.930
HDL cholesterol (mmol/l)	1.34 ± 0.41	1.27 ± 0.33	0.195
Triglycerides (mmol/l)	2.09 ± 1.47	2.07 ± 1.26	0.926
Hs-CRP (mg/l)*	3.5 (1.4-7.5)	2.8 (1.4-6.9)	0.607
Osteoprotegerin (μg/l)*	1.47 (1.02-1.89)	1.08 (0.85-1.40)	< 0.001

	**Myocardial ischemia**	**No myocardial ischemia**	
**Co-variables**	**(n = 92)**	**(n = 213)**	**p**

Blood pressure, systolic (mmHg)	141 ± 18	138 ± 18	0.117
Blood pressure, diastolic (mmHg)	81 ± 9	79 ± 11	0.164
HbA1c (%)	7.5 ± 1.4	7.2 ± 1.2	0.064
U-albumin creatinine ratio (mg/mmol)*	1.1 (0.0-4.0)	0.6 (0.0-3.3)	0.435
Total cholesterol (mmol/l)	4.3 ± 1.0	4.4 ± 1.0	0.750
LDL cholesterol (mmol/l)	2.2 ± 0.8	2.2 ± 0.8	0.931
HDL cholesterol (mmol/l)	1.23 ± 0.32	1.30 ± 0.35	0.118
Triglycerides (mmol/l)	2.09 ± 1.14	2.07 ± 1.36	0.924
Hs-CRP (mg/l)*	4.1 (1.3-7.4)	2.8 (1.5-6.4)	0.419
Osteoprotegerin (μg/l)*	1.11 (0.87-1.55)	1.11 (0.89-1.43)	0.711

Data are means ± SD or median (interquartile range). * tested with the Mann-Whitney nonparametric test.

**Table 3:** Association between risk factors of cardiovascular disease and different cardiovascular disease manifestations

Carotid arterial disease						
**Co-variables**		**Univariate**			Multivariate*	
	**Odds ratio**	**95% CI**	**p**	**Odds ratio**	**95% CI**	**P**

Blood pressure, systolic (mmHg)	1.01	1.00-1.03	0.030	1.01	1.00-1.03	0.102
Blood pressure, diastolic (mmHg)	1.00	0.97-1.02	0.648	1.00	0.99-1.03	0.391
Total cholesterol (mmol/l)	0.98	0.79-1.22	0.861	1.00	0.79-1.26	0.998
LDL cholesterol (mmol/l)	1.01	0.77-1.33	0.930	1.07	0.80-1.44	0.627
HDL cholesterol (mmol/l)	1.55	0.80-3.02	0.195	0.81	0.38-1.72	0.590
Triclycerides (mmol/l)	0.89	0.74-1.08	0.238	0.97	0.80-1.18	0.741
Hs-CRP (mg/l)	1.00	0.97-1.03	0.890	1.00	0.97-1.03	0.827
Osteoprotegerin (μg/l)	3.37	2.02-5.65	< 0.001	2.12	1.22-3.67	0.008
**Peripheral arterial disease**						

**Co-variables**		**Univariate**			Multivariate*	
	**Odds ratio**	**95% CI**	**p**	**Odds ratio**	**95% CI**	**P**

Blood pressure, systolic (mmHg)	1.02	0.99-1.03	0.066	1.01	0.99-1.03	0.237
Blood pressure, diastolic (mmHg)	0.97	0.93-1.00	0.033	0.97	0.94-1.00	0.058
Total cholesterol (mmol/l)	0.99	0.72-1.34	0.929	0.99	0.72-1.35	0.935
LDL cholesterol (mmol/l)	0.85	0.57-1.27	0.420	0.86	0.58-1.28	0.454
HDL cholesterol (mmol/l)	1.76	0.75-4.16	0.197	1.29	0.48-3.44	0.617
Triclycerides (mmol/l)	1.01	0.79-1.29	0.926	1.08	0.84-1.39	0.552
Hs-CRP (mg/l)	1.01	0.97-1.05	0.578	1.01	0.97-1.04	0.746
Osteoprotegerin (μg/l)	2.78	1.65-4.68	< 0.001	1.77	0.97-3.22	0.063
**Myocardial ischemia**						

**Co-variables**		**Univariate**			Multivariate*	
	**Odds ratio**	**95% CI**	**p**	**Odds ratio**	**95% CI**	**P**

Blood pressure, systolic (mmHg)	1.01	0.99-1.02	0.119	1.01	1.00-1.03	0.132
Blood pressure, diastolic (mmHg)	1.02	0.99-1.04	0.166	1.01	0.99-1.03	0.395
Total cholesterol (mmol/l)	0.96	0.76-1.22	0.749	0.96	0.75-1.22	0.714
LDL cholesterol (mmol/l)	1.01	0.75-1.36	0.931	1.01	0.75-1.36	0.960
HDL cholesterol (mmol/l)	0.54	0.25-1.17	0.119	0.65	0.29-1.48	0.306
Triclycerides (mmol/l)	1.01	0.84-1.22	0.924	0.97	0.80-1.18	0.749
Hs-CRP (mg/l)	1.02	0.99-1.05	0.256	1.02	0.99-1.05	0.289
Osteoprotegerin (μg/l)	1.13	0.73-1.77	0.584	1.26	0.77-2.06	0.364

Data are odds ratios, 95% confidence intervals (CI), and p-values. The outcome variables are carotid arterial disease, peripheral arterial disease, and myocardial ischemia, respectively.

*Adjusted for age, HbA1c and U-albumin-creatinine ratio.

## Discussion

The present study demonstrated in a large population of type 2 diabetes patients with no history of cardiovascular disease and relative short diabetes duration that plasma OPG levels were significantly increased in those with carotid arterial disease and PAD compared to patients without these manifestations. This was, however, not the case for patients with subtle signs of myocardial ischemia versus those without. When adjusted for age, HbA1c and U-albumin creatinine ratio in a multivariate logistic regression analysis, plasma OPG levels was associated with carotid arterial disease, but not with PAD or myocardial ischemia.

OPG is an important regulating molecule in bone turnover, and plasma OPG has been shown to correlate to bone and arterial diseases [21]. Of note, plasma OPG has been demonstrated to be an independent risk factor for the 10-year incidence of CVD and vascular mortality [22]. In diabetes mellitus, accumulation of OPG may be part of the generalized matrix changes seen in the arterial wall [9,23], which could relate to the fact that production of OPG from vascular smooth muscle cells is highly influenced by pro-inflammatory and hormonal factors in the diabetic milieu [10]. An observational study found higher plasma OPG concentrations in diabetes individuals compared with non-diabetics, although the absolute concentration difference was limited [21]. Therefore, it was highly relevant to investigate any relation between different CVD manifestations in type 2 diabetes patients and plasma OPG.

Our findings are compatible with the study by Ishiyama et al., who found that CIMT was positively correlated to plasma OPG levels in patients with type 2 diabetes [24]. Similarly, it has been demonstrated that the presence of carotid plaques [22] or increased CIMT [25], is well correlated to plasma OPG levels in healthy individuals. Altogether, we therefore find that the relation between plasma OPG and carotid arterial disease in type 2 diabetes patients is well-established. Of note, it has recently been published that CIMT can be determined with good and comparable reproducibility in type 2 diabetes patients as well as in non-diabetics [26].

Few studies have investigated the relation between PAD and plasma OPG levels. In these investigations PAD was associated with plasma OPG [27], but no study has, up till now, investigated the relationship between PAD and plasma OPG in type 2 diabetes patients. In the present study, we observed that plasma OPG was significantly increased in patients with PAD compared to patients without (Figure 1). Further, plasma OPG was associated with PAD in our type 2 diabetes patients in univariate logistic regression analysis, but not after adjusting for age, HbA1c and U-albumin creatinine ratio. The association between plasma OPG levels and both carotid arterial disease and PAD, respectively, expand earlier observations between plasma OPG and macrovascular disease [28].

The relationship between plasma OPG concentrations and signs of myocardial ischemia on MPS in patients with type 2 diabetes has previously been investigated by Avignon et al. Our data could, however not demonstrate this association. The study by Avignon et al. demonstrated that myocardial ischemia was associated with the upper quartile of plasma OPG levels [6], however, when comparing the two studies, they differ in at least five important ways, as 1) Avignon et al. included patients with two or more risk factors of CVD, whereas we had no exclusion criteria regarding the number of risk factors, 2) they included patients with known PAD (10%), whereas these were excluded in our study, 3) they included type 1 diabetes patients (24%) as well as type 2 diabetes patients (76%), whereas we exclusively included patients with type 2 diabetes, 4) the duration of diabetes was almost four times longer in their population compared to ours, and 5) finally they did not examine for carotid or peripheral arterial disease as we did. In conclusion, the patients in the study by Avignon et al. would properly have a higher a priori burden of atherosclerosis, which could to some extend explain why we could not find the same relation between myocardial ischemia and plasma OPG levels in our population of exclusive type 2 diabetes patients. The reason for the lack of relationship between myocardial ischemia on MPS and plasma OPG could be caused by the fact that MPS-results reflect relative flow differences in the coronary vessels, which can be decreased by either epicoronary artery stenosis or microvascular coronary disease. The epicoronary artery stenosis is caused by atherosclerosis, which is shown to correlate well with plasma OPG levels in type 2 diabetes [9]. However, microvascular coronary disease could be caused by endothelial dysfunction, something that is observed more frequently in type 2 diabetes patients both with and without coronary artery disease [29], and therefore from a theoretically point of view this should have no direct relationship to plasma OPG levels. For this reason, MPS could be consider as a less optimal investigation technique when investigating exclusively for coronary atherosclerosis, however, MPS was the only noninvasive technique available at the time, and we could not from a clinical point of view justify investigating our patients invasively.

A recent study by Altinova et al. investigated 166 consecutively enrolled type 2 diabetes patients, and found that plasma OPG was associated with age, glycemic control and microalbuminuria [30]. The study, however, did not state the number of patients with already known cardiovascular disease. Further the study did not examine each patient for cardiovascular disease in the three arterial vascular territories, where cardiovascular disease is often encountered, i.e., the coronary, carotid, and lower-extremity arteries. Other studies in type 2 diabetes patients have found that cardiovascular events were individually associated with increased age, dysregulated diabetes or microalbuminuria [31,32]. Therefore it seems reasonable that type 2 diabetes patients with increased age, dysregulated diabetes or microalbuminuria would have increased cardiovascular disease and therefore increased OPG levels compared with younger, well-controlled type 2 diabetes patients without microalbuminuria. For these reasons it is difficult to conclude from the study by Altinova et al. if plasma OPG levels were associated with diabetes related parameters (i.e. HbA1c and microalbuminuria) or these associations really were due to atherosclerosis [30]. In our study we found that plasma OPG was independently associated with carotid arterial disease when adjusting for age, HbA1c, and U-albumin creatinine ratio (adjusted OR: 2.12; 95% CI: 1.22-3.67; p = 0.008). Whereas peripheral arterial disease and myocardial ischemia were not when adjusting for the above mentioned parameters (adjusted OR: 1.77; 95% CI: 0.97-3.22; p = 0.063 and adjusted OR: 1.26; 95% CI: 0.77-2.06; p = 0.364). The reason for this could be that plama OPG levels were both associated to diabetes related parameters (i.e. HbA1c and U-albumin creatinine ratio) as well as to atherosclerotic related parameters (i.e. carotid arterial disease).

Our finding of associations between plasma OPG and atherosclerotic disease manifestations in type 2 diabetes is in agreement with the notion that this molecule may be involved in atherogenesis. The precise role of OPG in atherogenesis is unknown, however one suggestion is that the molecule function as a calcification inhibitor [11]. It is present in atherosclerotic plaques, particularly in areas with calcification [33], and may be compensatory upregulated in the atherogenic process. Other possible connections to atherosclerosis may, however also occur, since OPG has been shown to enhance the matrix content in plaques [34], and to be involved in endothelial function [35].

## Limitations

The present study has some potential limitations: 126 out of 431 eligible patients refused to participate or did not show up. However, no statistical differences were present between the study group and the non-participants with regard to age and diabetes duration (age: 58.6 ± 11.3 versus 60.7 ± 14.7 years, p = 0.15 and diabetes duration: 4.5 ± 5.3 versus 4.0 ± 6.5 years, p = 0.51). We did not test for other differences and, hence, selection bias cannot be totally excluded.

## Conclusions

In a consecutive series of type 2 diabetes patients without known or suspected CVD referred to a diabetes clinic for the first time with less than five years' average diabetes duration the present study found that plasma OPG levels were associated with the presence of carotid and peripheral arterial disease. Further, plasma OPG levels were not related to myocardial ischemia on MPS. When adjusting for age, HbA1c and U-albumin creatinine ratio plasma OPG levels were only associated with carotid arterial disease.

## List of abbreviations

ABI: ankle-brachial index; CIMT: carotid intima-media thickness; CVD: cardiovascular disease; MPS: myocardial perfusion scintigraphy; OPG: osteoprotegerin; PAD: peripheral arterial disease; RANK-L: receptor activator of nuclear factor-B ligand; TRAIL: TNF-related apoptosis inducing ligand; TSPI: toe systolic blood pressure.

## Authors' contributions

MKP carried out the clinical study (performed the ultrasound scans of the carotid arteries, strain gauge measurement and the myocardial scintigraphies). MKP, AJ, PFHC and LMR analysed the data. MKP and JEH participated in the design of the study and performed the statistical analysis. MKP and JEH drafted the manuscript. MN, JD, SEH, TSP, AJ, PFHC, HBN and LMR conceived of the study, and participated in its design and coordination and helped to draft the manuscript. All authors read and approved the final manuscript.

## Competing interests

The authors declare that they have no competing interests.
